# First Genome-Wide Association Study on Anxiety-Related Behaviours in Childhood

**DOI:** 10.1371/journal.pone.0058676

**Published:** 2013-04-02

**Authors:** Maciej Trzaskowski, Thalia C. Eley, Oliver S. P. Davis, Sophia J. Doherty, Ken B. Hanscombe, Emma L. Meaburn, Claire M. A. Haworth, Thomas Price, Robert Plomin

**Affiliations:** 1 MRC Social, Genetic and Developmental Psychiatry Centre, Institute of Psychiatry, King's College London, De Crespigny Park, London, United Kingdom; 2 Birkbeck, Department of Psychological Sciences, University of London, London, United Kingdom; University of Chicago, United States of America

## Abstract

**Background:**

Twin studies have shown that anxiety in a general population sample of children involves both domain-general and trait-specific genetic effects. For this reason, in an attempt to identify genes responsible for these effects, we investigated domain-general and trait-specific genetic associations in the first genome-wide association (GWA) study on anxiety-related behaviours (ARBs) in childhood.

**Methods:**

The sample included 2810 7-year-olds drawn from the Twins Early Development Study (TEDS) with data available for parent-rated anxiety and genome-wide DNA markers. The measure was the Anxiety-Related Behaviours Questionnaire (ARBQ), which assesses four anxiety traits and also yields a general anxiety composite. Affymetrix GeneChip 6.0 DNA arrays were used to genotype nearly 700,000 single-nucleotide polymorphisms (SNPs), and IMPUTE v2 was used to impute more than 1 million SNPs. Several GWA associations from this discovery sample were followed up in another TEDS sample of 4804 children. In addition, Genome-wide Complex Trait Analysis (GCTA) was used on the discovery sample, to estimate the total amount of variance in ARBs that can be accounted for by SNPs on the array.

**Results:**

No SNP associations met the demanding criterion of genome-wide significance that corrects for multiple testing across the genome (p<5×10^−8^). Attempts to replicate the top associations did not yield significant results. In contrast to the substantial twin study estimates of heritability which ranged from 0.50 (0.03) to 0.61 (0.01), the GCTA estimates of phenotypic variance accounted for by the SNPs were much lower 0.01 (0.11) to 0.19 (0.12).

**Conclusions:**

Taken together, these GWAS and GCTA results suggest that anxiety – similar to height, weight and intelligence − is affected by many genetic variants of small effect, but unlike these other prototypical polygenic traits, genetic influence on anxiety is not well tagged by common SNPs.

## Introduction

Anxiety disorders are among the most common psychiatric disorders [1]. They often begin in childhood [2] and continue into adulthood [3], when they become co-morbid with other psychiatric disorders especially depression [4] and entail significant costs both to society and to the individual [5]. Quantitative anxiety-related traits, assessed as clinical symptoms, e.g. [6] or personality/temperament traits [7], [8], are strong predictors of diagnosed anxiety disorders [7].

Twin studies have shown that childhood anxiety in representative samples, like other complex traits, is influenced genetically, e.g. [9]. Multivariate genetic studies indicate genetic overlap as well as specificity between different aspects of anxiety and from age to age as early as the preschool years [10] and into middle childhood [11] and adolescence [12], [13]. At age 7, the age of the twins in the present study, parent ratings of anxiety-related traits have been shown to be moderately heritable with both domain-general and trait-specific genetic effects [11]. Similar results were found at age 9 and for continuity from age 7 to age 9 [14]. Although these quantitative genetic findings are important, the next step is to identify specific genes responsible for these effects.

Until recently, molecular genetic investigation into the aetiology of anxiety relied on linkage and candidate-gene designs. Linkage, which looks for co-inheritance between DNA variants and a disorder within families, is a systematic strategy for detecting genes of large effect size throughout the genome. However, linkage found few such large effects for common disorders like anxiety and lacks power to detect more modest effects [15].

In contrast, allelic association, which looks for correlations between an allele and a trait among unrelated individuals, is much more powerful than linkage, but until recently, association has been limited to the exploration of a few candidate genes and could not be used to conduct a systematic search of the genome. Candidate-gene association studies of anxiety-related traits reported many associations but few of these associations have stood the test of replication, similar to candidate-gene studies in other domains in the life sciences [16].

Association studies became systematic with the advent of genome-wide DNA arrays that genotype hundreds of thousands of DNA variants throughout the genome and resulted in a plethora of genome-wide association (GWA) studies [17]. Although the first major GWA studies were reported in 2007 [18], significant results have been reported for more than 200 traits in 1500 GWA studies [19]. The only GWA studies of anxiety-related traits have focused on the personality trait of neuroticism in adults and reported possible associations with several genes [20], [21], [22]. However, no GWA studies of anxiety-related traits in children have previously been reported.

The current study presents the first GWA study of anxiety-related traits in children. The multivariate genetic results mentioned earlier led us to consider trait-specific as well as domain-general measures. Despite the success of GWA, reported associations are of small effect size and together account for only a modest proportion of the heritability of traits, known as the “missing heritability” problem [23], [24]. One of many possible reasons for the missing heritability problem is that potential associations are missed by the common SNPs that are included in extant DNA arrays. To test this hypothesis, a new technique, described by Yang et al. [25] and implemented in a software package called *Genomewide Complex Trait Analysis (GCTA)*, has been developed that allows estimation of the total genetic variance captured by SNPs on a genome-wide DNA array, even though it does not identify which SNPs are responsible for the genetic influence [26]. For this reason, we also report GCTA results for anxiety-related traits in childhood and compare them to our twin study estimates of heritability from the same sample at the same age and using the same measures.

## Methods

### Ethics Statement

Written parental consent was obtained prior to data collection and the project received approval from the Institute of Psychiatry ethics committee (05/Q0706/228).

### Sample

The sample was drawn from the Twins Early Development Study (TEDS), a multivariate longitudinal study which recruited over 11,000 twin pairs born in England and Wales in 1994, 1995 and 1996 [27], whose families are representative of the UK population [28]. Twins with severe medical problems or severe birth complications or whose zygosity could not be determined were excluded from the sample. To decrease heterogeneity of ancestry, the sample was restricted to families who identified themselves as white and whose first language was English. After exclusions, 7834 pairs of twins had anxiety data available at age 7 (mean age  = 7.06, SD  = 0.25). Although anxiety data were also available at age 9, we did not use these data in our GWA analyses because only half the sample were contacted at age 9 to provide phenotypic data.

3747 DNA samples from unrelated children in TEDS were sent for DNA array genotyping at the Wellcome Trust Sanger Institute, Hinxton, UK as part of the Wellcome Trust Case Control Consortium 2.

3665 samples were successfully hybridized to Affymetrix GeneChip 6.0 SNP genotyping arrays using standard experimental protocols (see Text S1). 3152 samples (1446 males and 1706 females) survived stringent quality control procedures performed (see Text S1), of whom 2810 also had anxiety data.

The replication sample was also drawn from TEDS children for whom DNA and anxiety data were available but for whom genome-wide genotyping was not available. After quality control, both anxiety data and SNP genotyping were available for 4804 additional individuals. Of these, 2625 were unrelated children who were also unrelated to children in the discovery sample; for 1742 children, their fraternal co-twin was in the discovery sample, and for 437 children their fraternal co-twin was also in the replication sample.

### Anxiety-Related Behaviours Questionnaire (ARBQ)

Anxiety was rated by parents using the Anxiety-Related Behaviours Questionnaire (ARBQ) [10]. The ARBQ is a quantitative trait parent rating instrument for children in the general population rather than a diagnostic tool. It includes items that assess anxiety symptoms as well as aspects of anxiety-related personality. The items are best structured as four latent variables in childhood: negative affect, negative cognition, fear, and social anxiety [11]. In order to investigate domain-general genetic associations, we also constructed a general anxiety composite by summing the standardised scores for these four variables. The overall composite was crucial to produce a phenotypic measure that was free from any scale-specific error. In addition, combining standardised scores assured that none of the scales biased the composite. The ARBQ has been shown to have good construct validity, and high internal consistency [10]. In order to avoid the skew that occurs for behaviour problem measures, the five anxiety scores were quantile normalised (van der Waerden; ranks averaged for tied data) [29]. Although the distributional properties of these transformed scores are better, the correlation between the raw scores and the transformed scores varied from .80 to .98 and results were highly similar for the raw and transformed scores.

### Genotyping

Genome-wide genotyping was done on AffymetrixGeneChip 6.0 SNP genotyping array with additional ∼2.5 million SNPs imputed from HapMap 2 and 3 and WTCCC controls Details about genotyping and quality control are included in the Text S1. 13 SNPs for the top hits for the five anxiety-related scales from the discovery sample were genotyped in the replication sample of 4804 individuals using the Sequenom MassARRAY iPlex Gold® system (Sequenom, San Diego, USA). Three SNPs failed to meet quality control criteria, leaving 10 SNPs available for the replication stage.

### Statistical Analyses

#### Genome-wide association (GWA) analysis

Linear regression analyses were conducted using SNPTEST v2.0 [18] under an additive model, using a frequentist method that accounts for uncertainty of genotype information [30]. We included age, sex, cohort and eight eigenvectors representing population ancestry as covariates. Consolidation and summary of the GWA results was performed in R (www.r-project.org) [31].

The strongest association results from the GWA were selected for genotyping in the replication sample. Where imputed SNPs were in LD with genotyped SNPs, the genotyped SNPs were preferred. However, one especially promising imputed SNP (rs1113313) was also selected. The SNPs were selected that were not in linkage disequilibrium (LD) with each other.

Sequenom genotyping results for the replication sample were analysed using the same protocols and software as those in GWA analysis. We conducted analyses using the total replication sample as well as the subsample of individuals genetically unrelated to each other or to individuals in the discovery sample. Although this is somewhat unorthodox, power is crucial for replication and the total sample provides maximum power because it maximises sample size. If replication is found for the total sample, the replication may be biased because the sample is not completely independent of the discovery sample and more replication would be required for definitive proof of replication. However, if the results from the discovery sample do not replicate using the total sample, this is the strongest possible evidence of failure to replicate because our replication sample consists of a highly similar sample tested at exactly the same age using exactly the same measures.

#### Genome-wide Complex Trait Analysis (GCTA)

GCTA does not attempt to identify specific variants associated with traits. Instead, it uses chance genetic similarity among unrelated individuals across hundreds of thousands of SNPs to predict phenotypic similarity. We used the GCTA software package [25] to evaluate the amount of the phenotypic variance explained by the genetic information available from the Affymetrix 6.0 DNA array. Detailed explanation of the methodology and procedure is available from Yang et al. [26]. To remain consistent with the procedure outlined by the proponents of the software, we initially used all ∼700,000 genotyped SNPs to calculate a genetic relatedness matrix (GRM). However, GCTA results reported previously for height, weight and intelligence used the Illumina microarray, which was designed with specific focus on European ancestry, whereas the Affymetrix microarray was less ancestry specific. We found that by adding high-quality imputed SNPs (see ‘Genotyping’ section), thus increasing the number of SNPs to ∼1.7 million, brought our GCTA estimates in line with previously published estimates for height, weight and intelligence. Thus, we used the 1.7 million SNPs to estimate how much of the heritability as estimated by the classical twin method could be accounted for by the available genetic information.

## Results

### Genome-wide Association (GWA)

**Figure 1:**
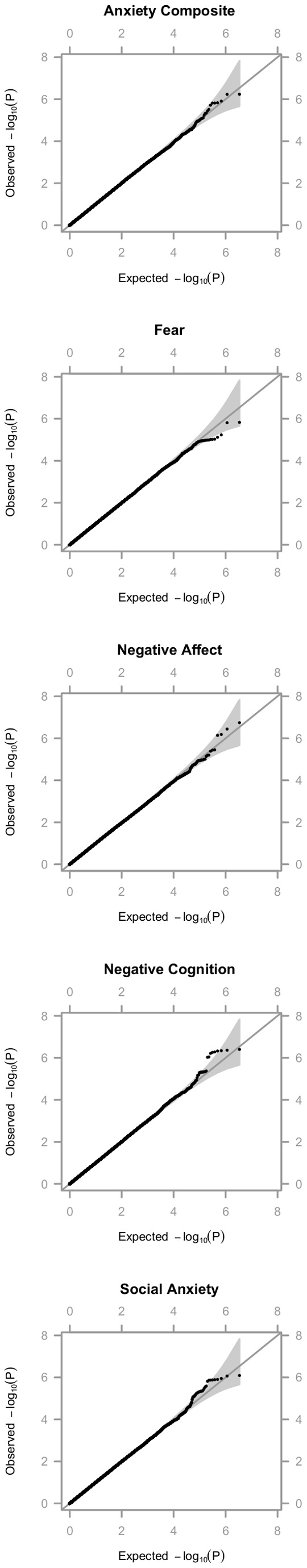
Log quantile-quantile (Q–Q) p-value plots for 1,724,317 single-SNP test of association of four anxiety-related traits and the anxiety composite at age 7. Footnote: Expected (X-axis) versus observed (Y-axis) p-values are plotted on the negative log scale to highlight the strongest associations. The diagonal line represents the null hypothesis and the grey polygons represent the 95% confidence interval (CI) of the null range. Significant association would be indicated by departure of the p-value (black dot) beyond the 95% CI of the null range.

Figure 1 presents quantile-quantile (Q-Q) plots for the five anxiety-related traits. Q–Q plots graphically compare the ∼1.7 million observed –log_10_ p values against the –log_10_ p values expected on the basis of no association. Although there is some increase of observed p values against expected p values for the lowest p values, few of the associations fall outside the 95% confidence bands (the grey areas), which indicates that there is little evidence of significant deviation from the null hypothesis of no association.

**Figure 2:**
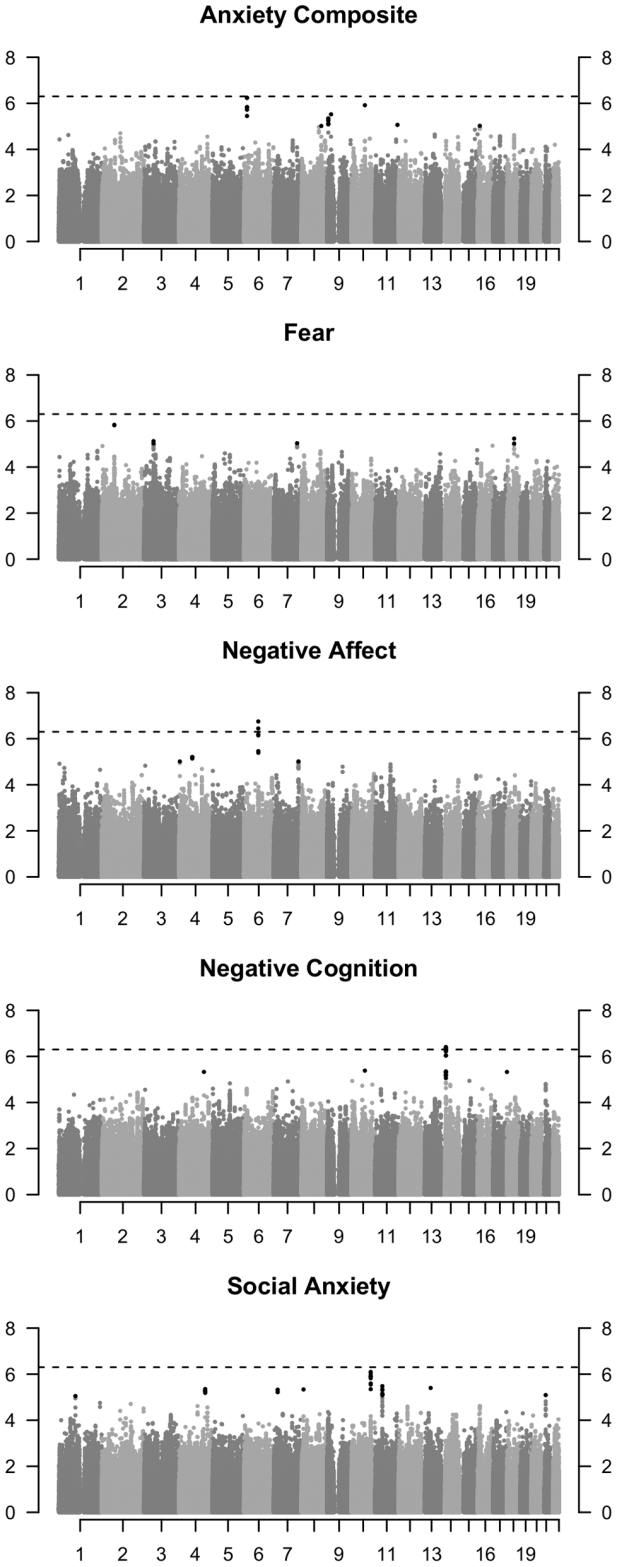
Manhattan plots for 1,724,317 single-SNP test of association for four anxiety-related traits and anxiety composite at age 7. Footnote: Oberved p-values are plotted on a scale of negative logs (Y-axis) against the SNP's physical position in the genome (X-axis). Black dots represent associations with p<5×10^−5^ and the horizontal dashed line represents suggestive significance with p<5×10^−7^.

Figure 2 presents ‘Manhattan’ plots for the same traits that show –log_10_ p values on the Y axis for the ∼1.7 million SNPs across the 22 autosomes on the X axis. The p values on the Y axis are the negative logarithms of the p values so that the highest points in the plot represent the strongest SNP associations. The dotted horizontal line represents suggestive significance (5×10^−7^), not genome-wide significance (5×10^−8^). Regions with the strongest associations were chosen for replication – for example, regions of chromosome 6 and 12 that reached suggestive significance (5×10^−7^) for the anxiety composite and negative cognition scale respectively. The association of the SNP on chromosome 6 with negative affect (Figure 2) was not proposed for replication due to the SNP's low minor allele frequency (maf  = 0.03). Table 1 shows results in the discovery sample for the 10 SNPs that were also successfully genotyped in the replication sample. Two of the lowest p values in the discovery sample were SNP rs16879771, associated with the anxiety composite (p = 6.27×10^−7^), and rs1952500, which was associated with Negative Cognition (p = 4.12×10^−7^). The significance of the remaining SNPs varied from 10^−4^ to 8−10^−7^. The amount of variance explained in the discovery sample as indicated by the squared beta values varied from 0.09% to 1.0%. Visual inspection of the genotype-specific means suggested that none of the selected SNPs deviated from additivity.

**Table 1:** Associations in the GWA discovery sample and in the replication sample for SNPs showing the lowest p values in the GWA analysis.

*rsid*	*chr*	*alleleA*	*alleleB*	*maf*	*gene*	*Phenotype*	*Beta discovery*	*Beta replication*	*P-value discovery*	*P-value replication*
*rs7649323*	3	C	G	0.36	DCP1A	Fear	0.06	0.02	7.42×10^−6^	0.11
*rs4568308*	4	A	G	0.21	EREG, BTC, AREG	Negative Affect	0.07	−0.003	6.73×10^−6^	0.28
*rs16879771*	6	C	T	0.08	CAP2	Anxiety Composite	−0.07	−0.03	6.27×10^−7^	0.15
*rs4130405*	8	A	C	0.15	NIPAL2, KCNS2	Anxiety Composite	0.05	0.01	1.70×10^−5^	0.40
*rs1113313*	10	C	T	0.41	VDAC2, SAMD8	Negative Cognition	−0.06	−0.04	4.20×10^−6^	0.44
*rs2772129*	10	A	G	0.38	SORCS1, XPNPEP1	Social Anxiety	0.07	−0.02	8.68×10^−7^	0.14
*rs10787217*	10	A	T	0.24	SORCS1, XPNPEP1	Negative Affect	−0.06	0.01	9.98×10^−5^	0.28
*rs2922037*	11	C	T	0.16	API5, LRRC4C	Social Anxiety	0.08	0.02	8.22×10^−6^	0.13
*rs1952500*	14	A	C	0.12	STXBP6, NOVA1	Negative Cognition	0.10	−0.06	4.12×10^−7^	0.13
*rs9977125*	21	C	T	0.38	TMPRSS15, C21orf131	Anxiety Composite	0.03	0.02	1.20×10^−4^	0.05

*Footnote: rsid – SNP id; chr – chromosome; maf – minor allele frequency; gene – nearest gene; p-values for the replication sample are one-tailed and uncorrected for multiple testing.*

### Replication

Table 1 also includes results for the 10 SNPs in the replication sample. None of the SNPs reached significance and the direction of the associations in the replication sample was nearly at a chance level (6 in the same direction as in the GWA analysis and 4 in the opposite direction). These replication analyses were based on our total replication sample of 4804 for which we had greatest power; similarly negative results were found for our subsample of 2625 individuals which constituted a more independent but less powerful replication sample.

### Genome-wide Complex Trait Analysis (GCTA)

As described earlier, we used ∼1.7 million SNPs to estimate the GCTA Genetic Relatedness Matrix for our sample of 2810 individuals. Our sample included no known pairs related in the traditional sense, which was confirmed by finding that no pairs reached the standard GCTA relatedness cut-off threshold of 0.025 genetic relatedness. Table 2 summarises the GCTA estimates obtained for the five anxiety-related traits and compares them to twin study heritability estimates from the sample at the same age using the same measures. Table 2 also includes GCTA estimates for height and weight in our sample in order to compare our results to previously reported results for height and weight. As indicated in Table 2, our twin study heritability estimates are 0.80 and 0.84 for height and weight, respectively, and our GCTA estimates are 0.35 and 0.42, all of which are comparable to results reported in the literature [32]. Also similar to the literature reviewed in the Introduction, our twin study heritabilities for anxiety-related traits are substantial, varying from 0.50 to 0.61. However, the GCTA estimates for anxiety-related traits were much lower, ranging from only 0.01 to 0.19. None of the GCTA estimates reached statistical significance (p<.05) due to the large standard errors of estimates.

**Table 2:** Genome-wide Complex Trait Analysis (GCTA) estimates of genetic variance compared to twin study estimates of heritability.

*Phenotype*	*GCTA estimate (SE)*	*P-value*	*Twin study h^2^ estimate (SE)*
***Negative Cognition***	0.07 (0.12)	0.257	0.52 (0.03)
***Negative Affect***	0.07 (0.12)	0.281	0.50 (0.03)
***Fear***	0.19 (0.12)	0.057	0.59 (0.02)
***Social Anxiety***	0.01 (0.11)	0.479	0.61 (0.01)
***Anxiety Composite***	0.16 (0.11)	0.075	0.52 (0.02)
***Height***	0.37 (0.14)	4×10^−4^	0.80 (0.02)
***Weight***	0.48 (0.14)	0.005	0.84 (0.02)

*Footnote: SE – standard error; n with non-missing phenotypic data  = 2806–2810 twin individuals (one co-twin per pair) for GCTA estimates and twin pairs for heritability estimates.*

## Discussion

This first genome-wide association study of anxiety-related traits in childhood indicates that no common genetic variants of large effect contribute to the heritability of these traits. Our sample of 2810 had 80% power to detect causal variants with effect sizes greater than 1.4% of the variance and none were detected. Power was calculated with the Genetic Power Calculator [33] using an additive model with a genome-wide significance threshold of p<5×10^−8^. As seen in Table 1, the largest effect size from the GWA analysis accounted for only 1% of the variance. Our power calculations indicated that we had less than 80% of power to detect a signal of this magnitude; thus this result should be considered with caution until replicated. That said, these results are similar to those found for other quantitative traits for which the strongest associations account for about 1% of the variance such as height [34], weight (GIANT Consurtium) [35], and cognitive traits including reading [36], mathematics [37], and general cognitive ability [38], [39]. Our GWA results for anxiety-related traits in childhood are compatible with a growing consensus from GWA studies of complex traits that the largest effect sizes are very small and that all known associations only explain a small portion of the heritabilities of complex traits and common disorders, a gap that is known as the *missing heritability problem* [23]. The missing heritability problem can be seen in Table 2 in which our twin study estimates of heritability for the five anxiety-related scales exceed 50%, whereas the sum of the effect sizes of the 10 SNPs shown in Table 2 is less than 5% in the discovery sample, and negligible in the replication sample.

Dozens of papers have been published about possible solutions to the missing heritability problem [40]. One possibility is that heritability might be overestimated in twin studies and another is that the common SNPs on commercially available DNA arrays might be missing associations due to very small effect sizes and also might be caused by rare polymorphisms of larger effect sizes [41]. Some of these issues are addressed in part by GCTA analysis. GCTA estimates overall genetic influence directly from overall SNP similarity pair by pair for a large population of unrelated individuals; in this sense, it is independent of the effect size of individual polymorphism, although it is limited to detecting the additive effects of the DNA array's common SNPs and the variants they tag. The large standard errors (Table 2) from our GCTA estimates based on a sample of 2810 indicate the daunting demands for power in trying to detect a tiny genetic signal from the noise of 1.7 million SNPs: Most of the population differ by less than 1% in overall SNP similarity across more than a million SNPs [42]. Nonetheless, for height and weight, our GCTA estimates are similar to those reported in the literature, which account for about half the heritability of these ‘anchor’ variables [32]. In contrast, across the five anxiety-related traits, the average GCTA estimate of 10% (Table 2) is less than one-fifth of the average twin-study heritability estimate of 55%. The total composite showed the highest, albeit non-significant, GCTA estimate but even this estimate was only about 30% of the twins study heritability estimate, which fell below the expected 50%. Importantly, consideration of the standard errors shows that if the SNPs accounted for 50% of the twin study heritability, as has been found with the ‘anchor’ variables height and weight, the GCTA results would have been significant in our study.

Two hypotheses for explaining the gap between these anxiety-related GCTA estimates and twin-study estimates are that GCTA underestimates genetic influence or that twin studies overestimate genetic influence, although these are not mutually exclusive hypotheses. We know that GCTA underestimates genetic influence to some extent because it only captures causal variants that are in linkage disequilibrium with the common SNPs used in the analysis; it misses the effect of rarer DNA variants not tagged by these SNPs. In addition, GCTA only assesses additive genetic effects. So, one possibility is that anxiety is influenced by rarer DNA variants or nonadditive genetic effects to a greater extent than height and weight.

On the other hand, our twin study heritability estimates for parental ratings may be inflated – most estimates of heritability of anxiety traits in childhood and adolescence using other assessment techniques are around 30% [43], which would put our GCTA estimates more nearly in range of accounting for half the heritability. Another possibility is that, unlike in GCTA, non-additive genetic variance can inflate estimates of additive genetic variance in a twin study. That is because its estimation is generally weak without extended family data [44].

It is important to resolve this issue of the gap between GCTA and twin-study estimates of heritability in general and specifically in terms of the possibility that the gap might be larger for anxiety-related traits than for other complex traits. To the extent that GCTA estimates account for heritability, it should be possible to identify genes responsible for the heritability of anxiety using common SNPs alone if samples are sufficiently large. Larger samples could result in closing this gap by producing an increased number of significant SNP associations in GWA and by providing GCTA estimates with smaller error terms. That said, a recent study reported GCTA estimate of 0.06(0.03) for neuroticism in a sample of nearly 12,000 adults [40]. Suggesting that this gap might remain opened until either data from exome-sequencing microarrays are available (that tag rarer variants), or until whole-genome sequencing identifies all variants of any kind [45].

### Conclusion

Our GWA results for anxiety-related traits suggest that, similar to other quantitative traits and common disorders, heritability is caused by many genes of small effect. Our GCTA results suggest that the genetic architecture of parent-rated anxiety-related traits may differ from previously published results in showing a greater gap between GCTA estimates of genetic influence and twin study estimates of heritability. One implication of knowing that there are no genes of large effect and that at least some of the genetic variance can be accounted for by the common SNPs on current DNA arrays is to increase sample sizes to detect associations of small effect size. Eventually, polygenic prediction, using composites of hundreds or thousands of DNA markers, may reach levels of predictive power useful at least for research if not for clinical practice.

## Supporting Information

Text S1 **Genotyping protocol, quality control and statistical analysis.**
(DOC)

Table S1 **Lambda Inflation rates for all variables.**
(DOCX)

## References

[1] Last CG, Perrin S, Hersen M, Kazdin AE (1992) DSM-III-R anxiety disorders in children: sociodemographic and clinical characteristics. J Am Acad Child Adolesc Psychiatry 31: 1070–1076. 1429407 10.1097/00004583-199211000-00012

[2] Kessler RC, Berglund P, Demler O, Jin R, Merikangas KR, et al (2005) Lifetime Prevalence and Age-of-Onset Distributions of DSM-IV Disorders in the National Comorbidity Survey Replication. Archives of General Psychiatry 62: 593–602. 15939837 10.1001/archpsyc.62.6.593

[3] Ferdinand RF, Dieleman G, Ormel J, Verhulst FC (2007) Homotypic versus heterotypic continuity of anxiety symptoms in young adolescents: evidence for distinctions between DSM-IV subtypes. Journal of Abnormal Child Psychology 35: 325–333. 17226094 10.1007/s10802-006-9093-0 PMC1915634

[4] Silberg JL, Rutter M, Eaves L (2001) Genetic and environmental influences on the temporal association between earlier anxiety and later depression in girls. Biological Psychiatry 49: 1040–1049. 11430845 10.1016/s0006-3223(01)01161-1

[5] Marciniak MD, Lage MJ, Dunayevich E, Russell JM, Bowman L, et al (2005) The cost of treating anxiety: The medical and demographic correlates that impact total medical costs. Depression and Anxiety 21: 178–184. 16075454 10.1002/da.20074

[6] Spence SH (1997) Structure of anxiety symptoms among children: a confirmatory factor-analytic study. Journal of Abnormal Psychology 106: 280–297. 9131848 10.1037//0021-843x.106.2.280

[7] Gladstone GL, Parker GB, Mitchell PB, Wilhelm KA, Malhi GS (2005) Relationship between self-reported childhood behavioral inhibition and lifetime anxiety disorders in a clinical sample. Depression and Anxiety 22: 103–113. 16149043 10.1002/da.20082

[8] Goldsmith HH, Lemery KS (2000) Linking temperamental fearfulness and anxiety symptoms: a behavior-genetic perspective. Biological Psychiatry 48: 1199–1209. 11137060 10.1016/s0006-3223(00)01003-9

[9] Franic S, Middeldorp CM, Dolan CV, Ligthart L, Boomsma DI (2010) Childhood and adolescent anxiety and depression: beyond heritability. J Am Acad Child Adolesc Psychiatry 49: 820–829. 20643315 10.1016/j.jaac.2010.05.013

[10] Eley TC, Bolton D, O'Connor TG, Perrin S, Smith P, et al (2003) A twin study of anxiety-related behaviours in pre-school children. The Journal of Child Psychology and Psychiatry 44: 945–960. 14531577 10.1111/1469-7610.00179

[11] Hallett V, Ronald A, Rijsdijk F, Eley TC (2009) Phenotypic and genetic differentiation of anxiety-related behaviors in middle childhood. Depression and Anxiety 26: 316–324. 19194998 10.1002/da.20539

[12] Kendler KS, Gardner CO, Lichtenstein P (2008) A developmental twin study of symptoms of anxiety and depression: evidence for genetic innovation and attenuation. Psychological Medicine 38: 1567–1575. 18578897 10.1017/S003329170800384X PMC2734978

[13] Silberg J, Pickles A, Rutter M, Hewitt J, Simonoff E, et al (1999) The influence of genetic factors and life stress on depression among adolescent girls. Arch Gen Psychiatry 56: 225–232. 10078499 10.1001/archpsyc.56.3.225

[14] Trzaskowski M, Zavos HM, Haworth CM, Plomin R, Eley TC (2012) Stable genetic influence on anxiety-related behaviours across middle childhood. J Abnorm Child Psychol 40: 85–94. 21766214 10.1007/s10802-011-9545-z PMC3268971

[15] Risch N, Merikangas K (1996) The future of genetic studies of complex human diseases. Science 273: 1516–1517. 8801636 10.1126/science.273.5281.1516

[16] Tabor HK, Risch NJ, Myers RM (2002) Candidate-gene approaches for studying complex genetic traits: practical considerations. Nat Rev Genet 3: 391–397. 11988764 10.1038/nrg796

[17] Hirschhorn JN, Daly MJ (2005) Genome-wide association studies for common diseases and complex traits. Nat Rev Genet 6: 95–108. 15716906 10.1038/nrg1521

[18] WTCCC (2007) Genome-wide association study of 14,000 cases of seven common diseases and 3,000 shared controls. Nature 447: 661–678. 17554300 10.1038/nature05911 PMC2719288

[19] Hindorff LA, Junkins HA, Mehta JP, Manolio TA (2012) A Catalog of Published Genome-Wide Association Studies. National Human Genome Research Institute.

[20] Calboli FC, Tozzi F, Galwey NW, Antoniades A, Mooser V, et al (2010) A genome-wide association study of neuroticism in a population-based sample. PloS one 5: e11504. 20634892 10.1371/journal.pone.0011504 PMC2901337

[21] Shifman S, Bhomra A, Smiley S, Wray NR, James MR, et al (2008) A whole genome association study of neuroticism using DNA pooling. Molecular Psychiatry 13: 302–312. 17667963 10.1038/sj.mp.4002048 PMC4004964

[22] van den Oord EJ, Kuo PH, Hartmann AM, Webb BT, Moller HJ, et al (2008) Genomewide association analysis followed by a replication study implicates a novel candidate gene for neuroticism. Arch Gen Psychiatry 65: 1062–1071. 18762592 10.1001/archpsyc.65.9.1062

[23] Maher B (2008) Personal genomes: The case of the missing heritability. Nature 456: 18–21. 18987709 10.1038/456018a

[24] Manolio TA, Collins FS, Cox NJ, Goldstein DB, Hindorff LA, et al (2009) Finding the missing heritability of complex diseases. Nature 461: 747–753. 19812666 10.1038/nature08494 PMC2831613

[25] Yang J, Lee SH, Goddard ME, Visscher PM (2011) GCTA: a tool for genome-wide complex trait analysis. American Journal of Human Genetics 88: 76–82. 21167468 10.1016/j.ajhg.2010.11.011 PMC3014363

[26] Yang J, Benyamin B, McEvoy BP, Gordon S, Henders AK, et al (2010) Common SNPs explain a large proportion of the heritability for human height. Nature Genetics 42: 565–569. 20562875 10.1038/ng.608 PMC3232052

[27] Oliver BR, Plomin R (2007) Twins' Early Development Study (TEDS): A multivariate, longitudinal genetic investigation of language, cognition and behavior problems from childhood through adolescence. Twin Research and Human Genetics 10: 96–105. 17539369 10.1375/twin.10.1.96

[28] Kovas Y, Haworth CMA, Dale PS, Plomin R (2007) The genetic and environmental origins of learning abilities and disabilities in the early school years. Monographs of the Society for Research in Child Development 72: 1–144. 17995572 10.1111/j.1540-5834.2007.00439.x PMC2784897

[29] Lehmann EL (1975) Nonparametrics: statistical methods based on ranks. San Francisco: Holden-Day.

[30] Marchini J, Howie BN, Myers S, McVean G, Donnelly P (2007) A new multipoint method for genome-wide association studies via imputation of genotypes. Nature Genetics 38: 906–913. 10.1038/ng2088 17572673

[31] R Development Core Team (2011) R: A Language and Environment for Statistical Computing. 2.13.1 ed. Vienna, Austria.

[32] Plomin R, Haworth CMA, Meaburn EL, Price T, 2WTCCC, et al. (in press) Common DNA markers can account for more than half of the genetic influence on cognitive abilities. Psychological Science. 10.1177/0956797612457952 PMC3652710 23501967

[33] Purcell S, Cherny SS, Sham PC (2003) Genetic Power Calculator: design of linkage and association genetic mapping studies of complex traits. Bioinformatics 19: 149–150. 12499305 10.1093/bioinformatics/19.1.149

[34] Lango Allen H, Estrada K, Lettre G, Berndt SI, Weedon MN, et al (2010) Hundreds of variants clustered in genomic loci and biological pathways affect human height. Nature 467: 832–838. 20881960 10.1038/nature09410 PMC2955183

[35] Walley AJ, Asher JE, Froguel P (2009) The genetic contribution to non-syndromic human obesity. Nat Rev Genet 10: 431–442. 19506576 10.1038/nrg2594

[36] Meaburn EL, Harlaar N, Craig IW, Schalkwyk LC, Plomin R (2007) Quantitative trait locus association scan of early reading disability and ability using pooled DNA and 100K SNP microarrays in a sample of 5760 children. Mol Psychiatry 13: 729–740. 17684495 10.1038/sj.mp.4002063

[37] Docherty SJ, Kovas Y, Petrill SA, Plomin R (2010) Generalist genes analysis of DNA markers associated with mathematical ability and disability reveals shared influence across ages and abilities. BMC Genet 11: 61. 20602751 10.1186/1471-2156-11-61 PMC2909150

[38] Davies G, Tenesa A, Payton A, Yang J, Harris SE, et al (2011) Genome-wide association studies establish that human intelligence is highly heritable and polygenic. Mol Psychiatry 16: 996–1005. 21826061 10.1038/mp.2011.85 PMC3182557

[39] Davis O, Butcher L, Docherty S, Meaburn E, Curtis C, et al (2010) A Three-Stage Genome-Wide Association Study of General Cognitive Ability: Hunting the Small Effects. Behavior Genetics 40: 759–767. 20306291 10.1007/s10519-010-9350-4 PMC2992848

[40] Vinkhuyzen AA, Pedersen NL, Yang J, Lee SH, Magnusson PK, et al (2012) Common SNPs explain some of the variation in the personality dimensions of neuroticism and extraversion. Translational psychiatry 2: e102. 22832902 10.1038/tp.2012.27 PMC3337075

[41] McCarthy MI, Hirschhorn JN (2008) Genome-wide association studies: potential next steps on a genetic journey. Human molecular genetics 17: R156–165. 18852205 10.1093/hmg/ddn289 PMC2782356

[42] Plomin R (2012) Genetics: How intelligence changes with age. Nature 482: 165–166. 22318596 10.1038/482165a

[43] Eley TC, Gregory A.M. (2004) Behavioral genetics. Anxiety disorders in children and adolescents. New York: Guilford Press.

[44] Plomin R, DeFries JC, Knopik VS, Neiderhiser JM (2012) Behavioral Genetics. New York: Worth Publishers. 560 p.

[45] Plomin R (2012) Child Development and Molecular Genetics: 14 Years Later. Child Dev 30: 1467–8624. 10.1111/j.1467-8624.2012.01757.x PMC3593231 22469254

