# Correction: First Genome-Wide Association Study on Anxiety-Related Behaviours in Childhood

**DOI:** 10.1371/annotation/d320e3eb-ab8f-43ce-b341-3da36cd7ff99

**Published:** 2013-06-27

**Authors:** Maciej Trzaskowski, Thalia C. Eley, Oliver S. P. Davis, Sophia J. Doherty, Ken B. Hanscombe, Emma L. Meaburn, Claire M. A. Haworth, Thomas Price, Robert Plomin

The name of the fourth author was spelled incorrectly. The correct name is: Sophia J. Docherty. 

The correct citation is: Trzaskowski M, Eley TC, Davis OSP, Docherty SJ, Hanscombe KB, et al. (2013) First Genome-Wide Association Study on Anxiety-Related Behaviours in Childhood. PLoS ONE 8(4): e58676. doi:10.1371/journal.pone.0058676. 

