# Supplementary material for: First Genome-Wide Association Study on Anxiety-Related Behaviours in Childhood
Source: PLoS One. 2013 Apr 2;8(4):e58676. doi: 10.1371/journal.pone.0058676 (PMC3614558; doi:10.1371/journal.pone.0058676)
Supplement: Text S1 — Genotyping protocol, quality control and statistical analysis. (DOC) [file pone.0058676.s001.doc]

**Text S1. Genotyping protocol, quality control and statistical analysis**

**Genotyping protocol**

DNA was extracted from buccal cheek swabs and sent toAffymetrix, Santa Clara, California, USA. In total, 3,665 samples were successfully hybridized to AffymetrixGeneChip 6.0 SNP genotyping arrays (<http://www.affymetrix.com/support/technical/datasheets/genomewide_snp6_datasheet.pdf>) using experimental protocols recommended by the manufacturer (Affymetrix Inc., Santa Clara, CA). The raw image data from the arrays were normalized and pre-processed at the Wellcome Trust Sanger Institute, Hinxton, UK for genotyping as part of the Wellcome Trust Case Control Consortium 2 (<https://www.wtccc.org.uk/ccc2/>) according to the manufacturer’s guidelines (http://www.affymetrix.com/support/downloads/manuals/genomewidesnp6_manual.pdf). Genotypes for the Affymetrix arrays were called using CHIAMO (https://mathgen.stats.ox.ac.uk/genetics_software/chiamo/chiamo.html).

Where there was a sufficient quantity of DNA, samples were also re-genotyped on a panel of 30 SNPs (including 26 autosomal SNPs present on the Affymetrix array, and 4 SNPs on the X chromosome to verify gender) using the SequenomiPlex Gold assay (Sequenom Inc., San Diego, CA).

**Quality Control: Samples**

We identified and removed samples whose genome-wide patterns of diversity differed from those of the collection at large, interpreting these differences as likely to be due to biases or artifacts. Outlying individuals were identified on the basis of call rate, heterozygosity, relatedness and ancestry using a Bayesian clustering approach .

To obtain a set of putatively unrelated individuals we used a hidden Markov model (HMM) to infer identify by descent along the genome between pairs of individuals. Amongst pairs of closely related individuals, we excluded the member of the pair with the lowest call rate, iteratively repeating this procedure to obtain a set of individuals with pairwise identity by decent less than 5% .

Of the individuals genotyped, samples were excluded because of low call rate or heterozygosity outliers (377), unusual hybridization intensity (9), atypical population ancestry (59), sample duplication or relatedness to other sample members (83), and gender mismatches (13). In addition, 54 samples were excluded because fewer than 90% of genotypes were called identically on the genome-wide array and Sequenom panel. The remaining samples were consistent with previous genotyping. In total, 513 samples were excluded by these quality control criteria. The remaining sample of 3,152 individuals included 1,446 males and 1,706 females.

**Quality Control: SNPs**

A measure of information for the allele frequency at each of 932,533 called SNPs was calculated using SNPTEST version 2.1.1 . Autosomal SNPs were excluded if this information measure was below 0.975, if the minor allele frequency was less than 1%, if greater than 2% of genotype data were missing, or if the Hardy Weinberg *p*-value was lower than 10^-20^. Association between the SNP and the plate on which samples were genotyped was calculated; SNPs with a plate effect *p*-value less than 10^-6^ were also excluded. In addition, SNPs were manually filtered for call quality by visual inspection of the hybridization intensity plots using EVOKER software (<http://sourceforge.net/projects/evoker/>). The above filters removed 26 % of the SNPs, leaving 690,943 autosomal SNPs for further analysis.

**Statistical analysis**

Imputation was carried out using the IMPUTE version 2 software on the genotype data after application of quality control procedures, using a two-stage approach with both a haploid reference panel and a diploid reference panel. For the haploid reference panel we used HapMap phase II and III SNP data on the 120 unrelated CEU trios. 5,175 WTCCC2 controls were genotyped on both Affymetrix 6.0 and Illumina Human1.2M-Duo arrays (Illumina Inc., La Jolla, CA), and these were used for the diploid reference panel. SNPs were retained for analysis if they were genotyped using the Affymetrix 6.0 array, if they were genotyped using the Illumina Human1.2M-Duo array and obtained an information score ≥ 0.90, or if they were imputed and obtained an information score ≥ 0.98. Using these criteria, 1,033,462 imputed SNPs were retained giving a total of 1,724,405 SNPs used in the association analyses.

We performed Principal Component Analysis on a subset of 105,556 autosomal SNPs remaining after applying our quality control criteria, after pruning to remove SNPs in high linkage disequilibrium (*r*^2^> 0.2) and excluding high linkage disequilibrium genomic regions so as to ensure that only genome-wide effects were detected (Fellay et al., 2007). Application of the Tracy-Widom test indicated that eight principal components were significant using a threshold of *p*< 0.05. Phenotype scores were normalized by transforming the ranked data to the quantiles of a standard normal distribution using the van der Waerden transformation . Linear regression analysis was run for each autosomal SNP using a frequentist additive model that accounts for uncertain genotype data, as implemented in SNPTEST version 2.1.1 . Sex and the first eight principal components of the genotype data were included as covariates in the regression model.

SOM References

1. Barrett JC, Lee JC, Lees CW, Prescott NJ, Anderson CA, et al. (2009) Genome-wide association study of ulcerative colitis identifies three new susceptibility loci, including the HNF4A region. Nat Genet 41: 1330-1334. Epub 2009 Nov 1315.

2. Marchini J, Howie BN, Myers S, McVean G, Donnelly P (2007) A new multipoint method for genome-wide association studies via imputation of genotypes. Nature Genetics 38: 906-913.

3. Howie BN, Donnelly P, Marchini J (2009) A Flexible and Accurate Genotype Imputation Method for the Next Generation of Genome-Wide Association Studies. Plos Genetics 5: e1000529.

4. Patterson N, Price AL, Reich D (2006) Population structure and eigenanalysis. Plos Genetics 2: e190.

5. van der Waerden BL (1952) Order tests for the two-sample problem and their power. Indagationes Mathematicae: 453-458.
