# Supplementary material for: First Genome-Wide Association Study on Anxiety-Related Behaviours in Childhood
Source: PLoS One. 2013 Apr 2;8(4):e58676. doi: 10.1371/journal.pone.0058676 (PMC3614558; doi:10.1371/journal.pone.0058676)
Supplement: Table S1 — Lambda Inflation rates for all variables. (DOCX) [file pone.0058676.s002.docx]

**Table S1. Lambda Inflation rates for all variables**

|  | Genotyped SNPs | Imputed SNPs |
| --- | --- | --- |
| Negative Cognition | 0.997 | 1.001 |
| Negative Affect | 1.006 | 1.006 |
| Fear | 0.998 | 1.010 |
| Social Anxiety | 1.001 | 1.007 |
| Anxiety Composite | 1.006 | 1.006 |

Inflation rate was calculated separately for imputed and for genotyped SNPs. λ = median(x)/4.56.
